# Clinical prediction models of rheumatoid arthritis and its complications: focus on cardiovascular disease and interstitial lung disease

**DOI:** 10.1186/s13075-023-03140-5

**Published:** 2023-09-01

**Authors:** Yubo Shao, Hong Zhang, Qi Shi, Yongjun Wang, Qianqian Liang

**Affiliations:** 1 Longhua Hospital, Shanghai University of Traditional Chinese Medicine, Shanghai, 200032 China; 2 Spine Institute, Shanghai University of Traditional Chinese Medicine, Shanghai, 200032 China; 3 School of Graduate, Shanghai University of Traditional Chinese Medicine, Shanghai, 201203 China; 4 Key Laboratory of Ministry of Education of Theory and Therapy of Muscles and Bones, Shanghai University of Traditional Chinese Medicine, Shanghai, 200032 China

**Keywords:** Rheumatoid arthritis, Cardiovascular disease, Interstitial lung disease, Prediction models, Pathogenesis

## Abstract

Rheumatoid arthritis (RA) is a chronic, systemic, autoimmune disease of unknown etiology with erosive, symmetric polyarthritis as the main clinical manifestations. Its basic pathological changes are the formation of synovitis, and patients gradually develop destruction of articular cartilage destruction and bone erosion, which eventually leads to joint deformity, disability, and various extra-articular manifestations. Clinical prediction models (CPMs), also known as risk prediction models or risk scores, are mathematical formulas used to estimate the probability that a given individual will have a disease or an outcome in the future. The models are mainly divided into two categories: diagnostic models and prognostic models, which can be used to provide information on disease diagnosis or prognosis to help make better medical decisions. Currently, there is no cure for RA, but effective early diagnosis and treatment are crucial for limiting the severity of the disease and preventing the occurrence and development of complications. This paper reviews the CPMs associated with RA and its related complications, including cardiovascular disease (CVD) and interstitial lung disease (ILD), in order to provide reference and evidence for the early diagnosis and treatment of these diseases and personalized medicine for patients. In addition, the possible pathogenesis and risk factors of these comorbidities are summarized, and possible directions for future related research are prospected.

## Background

**Fig. 1:**
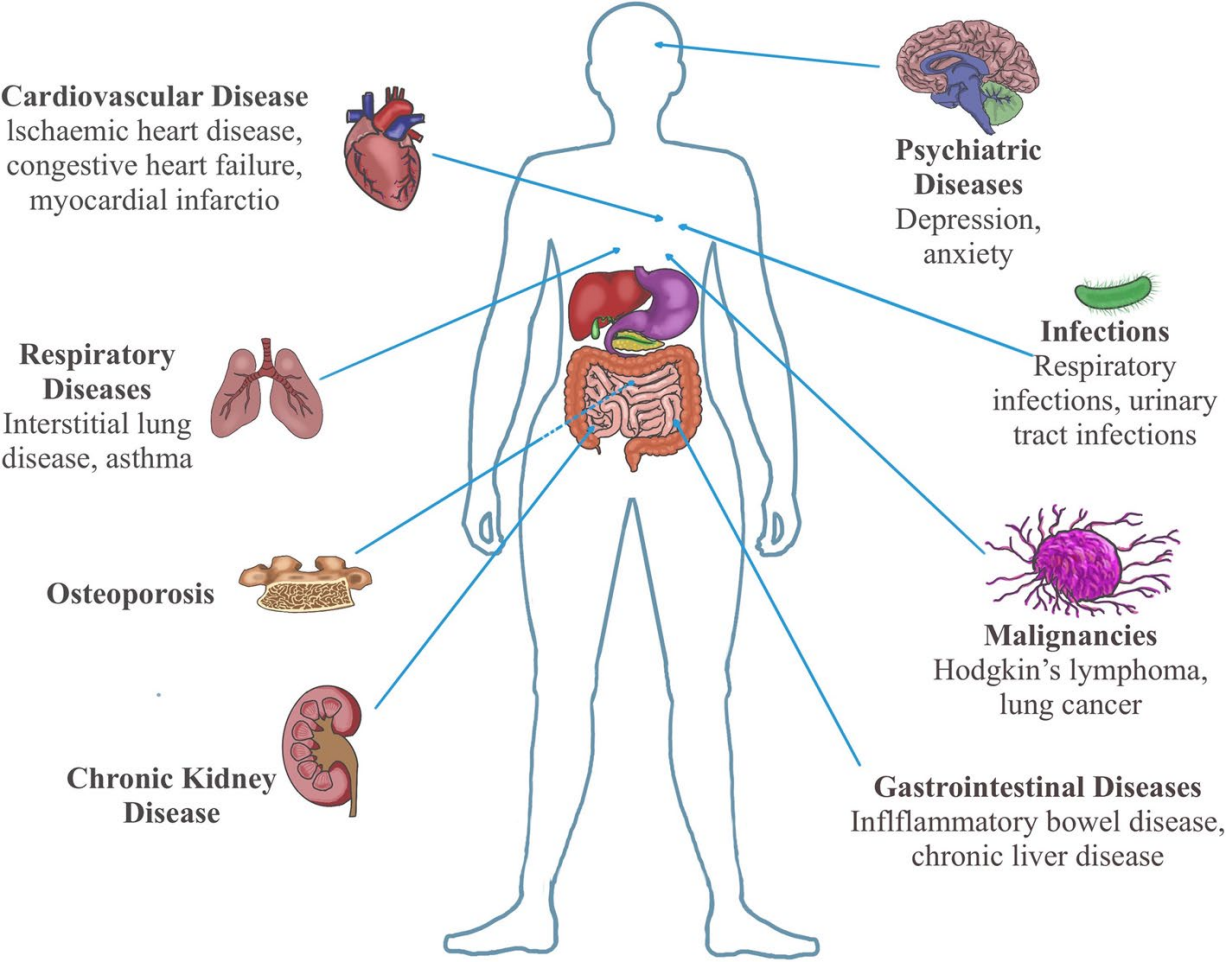
Schematic illustration of systemic complications of rheumatoid arthritis. The most frequent comorbidities of patients with rheumatoid arthritis include cardiovascular disease, respiratory diseases, infectious diseases, psychiatric diseases, gastrointestinal diseases, malignancies, chronic kidney disease, and osteoporosis

Rheumatoid arthritis (RA) is a chronic, systemic, autoimmune disease of unknown etiology with erosive, symmetric polyarthritis as the main clinical manifestations [1]. Its basic pathological changes are the formation of synovitis, and patients gradually develop destruction of articular cartilage destruction and bone erosion, which eventually leads to joint deformity, disability, and various extra-articular manifestations [2]. Chronic, persistent, and systemic inflammation in RA is characterized by an increase in specific inflammatory mediators, cytokines, and related antibodies, and a combination of genetic and environmental factors predisposes patients to different comorbidities and increases the risk of disability and death [3]. It is estimated that comorbidities are present in nearly 80% of inpatients with RA [4], such as cardiovascular disease (CVD), respiratory diseases including interstitial lung disease (ILD), infectious diseases, psychiatric diseases, gastrointestinal diseases, malignancies, chronic kidney disease, and osteoporosis (OP) et al. [3, 5] (Fig. 1). CVD is a prevalent complication of RA and represents the leading cause of mortality for patients [3]. Additionally, ILD is both the most common and severe manifestation of RA-related lung diseases [3]. The occurrence of these comorbidities will not only aggravate the condition of RA, but also further reduce the quality of life of patients and lead to a shortened life expectancy [2]. Currently, there is no cure for RA, and the goal of treatment is to maximize remission [6, 7]. Effective early diagnosis and treatment are of great significance to limit disease severity and prevent the occurrence and development of complications [3]. Therefore, in addition to new drug development and mechanism research, it is equally important to predict the effective response of RA patients to therapeutic drugs and early identification of patients who are prone to various complications.

When the medical model develops from empirical medicine through evidence-based medicine to precision medicine, the acquisition, storage, analysis, and prediction technology of medical data has developed rapidly, making the vision of personalized medicine more and more possible [8]. Clinical prediction models (CPMs), also known as risk prediction models or risk scores, are mathematical formulas used to estimate the probability that a given individual will have a disease or an outcome in the future [9], mainly divided into diagnostic models and prognostic models, which can be used to provide information on disease diagnosis or prognosis to help make better medical decisions. In recent years, there have been several studies reporting on CPMs related to RA, RA-CVD, and RA-ILD. However, there is a lack of comprehensive summaries regarding these models. This paper reviews the CPMs related to RA, RA-CVD, and RA-ILD, in order to provide reference and evidence for the early diagnosis and treatment of these diseases and personalized medicine for patients, and the possible pathogenesis and risk factors of RA-CVD and RA-ILD are summarized, and possible directions for future-related research are prospected.

## RA

### Models predicting the risk of RA

Due to the characteristics of RA that cannot be cured at present, treatment should be initiated as soon as possible once RA is diagnosed, as early treatment can significantly slow disease progression and prevent irreparable joint damage and disability [2, 10]. Therefore, identifying individuals at high risk for RA and making an early diagnosis are particularly important.

Karlson et al. [11] developed predictive models for RA (Table 1). These models were constructed using 8 human leucocyte antigen (HLA) alleles, 14 single nucleotide polymorphisms (SNPs), and clinical factors and generated an integrated, weighted genetic risk score (GRS) calculated as the product of individual-locus odds ratios. The model including genetic variables has a higher predictive ability than the model containing only clinical factors. The group went on to extend this research by incorporating 17 newly validated RA risk alleles into the GRS and assessing the GRS in relation to the more specific phenotypes of RA along the severity continuum [12] (Table 1). New models were able to forecast seronegative, seropositive, erosive and seropositive, and erosive RA, achieving area under the curve (AUC) values of 0.56, 0.65, 0.64, and 0.712 respectively. The results indicate that the GRS has virtually no ability to distinguish between the control group and seronegative RA, and the addition of 17 new alleles does not improve the predictive capability of the GRS. In contrast, this also suggests that seropositive RA and seronegative RA have distinct genetic foundations. Therefore, conducting separate studies on these two phenotypes in future research would provide a deeper comprehension of the genetic and functional composition of the disease.

**Table 1:** Summary of clinical prediction models of RA, RA-CVD, and RA-ILD

Study diseases	Study population/sample size	Predictors	Outcome measures	Model	Model representation	External validation	Reference
RA	American and Swedish/289–629	14 SNPs, 8 HLA alleles, age, gender, smoking	Occurrence of RA	Logistic regression	NR	Yes	Karlson/2010 [11]
RA	American/542	31 SNPs, 8 HLA alleles, smoking	Seronegative, seropositive, erosive, seropositive and erosive RA phenotypes	Logistic regression	NR	Yes	Chibnik/2010 [12]
RA	5 countries/11,366	45 RA non-HLA susceptibility loci, imputed amino acids at HLA-DRB1 (11, 71, and 74), HLA-DPB1 (position 9), HLA-B (position 9), gender	Occurrence of RA	Logistic regression	NR	Yes	Yarwood/2015 [13]
RA	American and Swedish/317–987	31 non-HLA alleles, 8 HLA-DRB1 alleles, HLA-SE*smoking, age, smoking, alcohol, education, parity, region, menopause, exposure to silica	Occurrence of RA	Logistic regression	NR	Yes	Karlson/2013 [14]
RA	American and Swedish/381–1244	31 non-HLA alleles, 8 HLA-DRB1 alleles, age, smoking, alcohol, education, parity, BMI, family history	Occurrence of RA	Logistic regression	NR	Yes	Sparks/2015 [10]
RA	England/80–2623	15 four-digit/10 two-digit HLA-DRB1 alleles, 31 SNPs, male ever-smoking status	Risk and age of onset of RA	COX regression	Diagram	No	Scott/2013 [15]
RA	Multiple ethnicities/8683	diabetes, depression, BMI, hypertension, gout, gender, ethnicity, smoking, sleep hours, income to poverty ratio	Occurrence of RA	Bayesian logistic regression	NR	Yes	Lufkin/2021 [16]
RA	Singaporean Chinese/599	9 SNPs	Occurrence of RA	Machine learning	Webpage	Yes	Lim/2023 [17]
RA	Netherlander/205	RF, gender, smoking status, DAS, AMPD1, ATIC, ITPA, MTHFD1	Insufficient response to MTX	Logistic regression	Risk score	Yes	Wessels/2007 [18]
RA	Netherlander/285	ABCB1 rs1045642 genotype, ABCC3 rs4793665 genotype, erythrocyte-folate, DAS28, HAQ, current smoking, BMI	Insufficient response to MTX	Logistic regression	Risk score	Yes	De Rotte/2018 [19]
RA	Japanese/134	SLCO3A1, CYP7A1, CHST10, GGH, SLC22A1, EPHX1, ATP7B, DAS28, folic acid	Efficacy and hepatotoxicity of MTX	Logistic regression	NR	No	Onishi/2020 [20]
RA	Netherlander/91	Erythrocyte-folate, DAS28, HAQ, current smoking, BMI	Insufficient response to MTX	Logistic regression	Webpage	No	Gosselt/2020 [21]
RA	Norway/218	Gender, SJC, RAI, PGA, ACPA, CRP, radiographic erosions, US, and MRI variables	Insufficient response to MTX and future structural damage progression	Logistic regression	NR	No	Sundin/2021 [22]
RA	40 countries/3280	Gender, HAQ, presence of comorbidities, age, TJC, ESR	Treatment response to golimumab	Logistic regression	Diagram	Yes	Vastesaeger/2016 [23]
RA	Netherlander/80	CD14+, T cells, CD4+ T cells, PBMC RNA, PBMC DNA	Treatment response to adalimumab or etanercept	Random forest	Diagram	Yes	Tao/2021 [24]
RA	Netherlander/93	DAS28, interferon score, DMARDs use	Treatment response to rituximab	Logistic regression	NR	Yes	De Jong/2018 [25]
RA	Multiple countries/164	Synovial biopsy-based gene expression and histological data	Response to rituximab, tocilizumab and multidrug resistance	Elastic net regression and GBM	NR	No	Rivellese/2022 [26]
RA-CVD	American/15,744	Age, sex, diabetes, hyperlipidemia, hypertension, tobacco use, CDAI, modified HAQ, prednisone use, RA duration	Occurrence of CVD events, including MI, stroke, or fatal CVD in the next 10 years	COX regression	Risk score	Yes	Solomon/2015 [27]
RA-CVD	American/20,467	MBDA score, age, diabetes, hypertension, tobacco use, CVD history, leptin, MMP-3, and TNF-R1	Occurrence of CVD events, including MI, stroke, or fatal CVD in the next 3 years	COX regression	Mathematical formula	No	Curtis/2020 [28]
RA-CHD	Chinese/1012	Age, hypertension, ACPA, LDL, HDL, TG, ESR	Occurrence of CHD	Logistic regression	Nomogram	Yes	Wei/2022 [29]
RA-ILD	Italians/90	VC	Occurrence of ILD	NR	NR	Yes	Pancaldi/2018 [30]
RA-ILD	Chinese/183	Male, smoke, cough, VC, have taken MTX, RF, ACPA, cold wet paralysis obstruction	Occurrence of ILD	Logistic regression	Nomogram	No	Ge/2021 [31]
RA-ILD	Japanese/58	FVC, PaO2/FiO2 ratio	mortality of after 90 days of AE-RA-ILD	Recursive partitioning	Decision tree	No	Hozumi/2022 [32]

*Abbreviations*: *ACPA* anti-citrullinated protein antibodies, *AE* acute exacerbation, *BMI *body mass index, *cfPWV* carotid-femoral pulse wave velocity, *CHD* coronary heart disease, *CRP* C-reactive protein, *CVD* cardiovascular disease, *CDAI* clinical disease activity index, *DAS28* disease activity score 28, *DMARDs* disease-modifying antirheumatic drugs, *ECG* electrocardiographic, *ESR* erythrocyte sedimentation rate, *FVC* forced vital capacity, *GBM* gradient-boosted machine, *GC* glucocorticoid, *HAQ* health assessment questionnaire, *HDL* high-density lipoprotein cholesterol, *HLA *human leucocyte antigen, *ILD* interstitial lung disease, *LDL* low-density lipoprotein cholesterol, *MBDA* multi-biomarker disease activity, *MI* myocardial infarction, *MMP* matrix metalloproteinase, *MRI *magnetic resonance imaging, *MTX* methotrexate, *NR* not reported, *PGA* patient global assessment, *RA* rheumatoid arthritis, *RAI* Ritchie articular index, *RF* rheumatoid factor, *SJC* swollen joint count, *SNPs* single nucleotide polymorphisms, *TG* triglyceride, *TJC* tender joint count, *TNF* tumor necrosis factor, *US* ultrasound, *US7* 7-joint ultrasonic erosions score, *VC* velcro crackle

Several other studies [10, 13, 14] (Table 1) have conducted comparable predictive analyses, utilizing a blend of clinical and genetic risk factors to devise models with good discriminative ability. A study [15] (Table 1) introduced a novel modeling approach, with model development facilitated by an R package [33]. This program incorporates published gene environment risk factor and disease statistics to categorize risk using a confidence interval (CI)-based approach within a simulated population. This study found that HLA and smoking status can be used to predict the risk of younger and older onset RA, respectively.

The involvement of genetic variables remains a double-edged sword, as it can enhance predictive capability on the one hand, but on the other hand, its difficulty in acquisition can hinder clinical application. A model devoid of genetic variables for RA has been developed, which solely utilizes common risk factors as predictors, including comorbidities, demographic, socioeconomic, and behavioral risk factors [16] (Table 1). In addition to delivering high predictive accuracy, the model has the ability to capture the impacts of individual variables along with the crucial higher-order interactions among them. For instance, age not only serves as a crucial predictor for RA, but it also exhibits strong interactive effects with variables such as smoking and depression.

Recently, an optimized polygenic risk score calculator using machine learning (ML) for RA was developed based on 9 ML-identified SNPs [17] (Table 1), which can be accessed through this link: https://xistance.shinyapps.io/prs-ra/ [17]. This model has extremely high predictive capability (AUC > 0.9), and it is very user-friendly. However, the fact that the derivation and validation data are both derived from the Singaporean Chinese underscores the need for continuous validation across different regions and ethnicities.

### Models predicting insufficient response to methotrexate (MTX)

Currently, methotrexate (MTX) is recommended as a first-line treatment for RA [6]; however, approximately one-third of patients do not respond sufficiently to this medication [34]. Identifying who are likely to have a suboptimal response to MTX treatment prior to initiating therapy could potentially lead to better initial treatment decisions for patients with RA.

A clinical pharmacogenetic model was to predict the efficacy of MTX in RA [18] (Table 1). A scoring system ranging from 0 to 11.5 has been developed for convenient clinical use. The model combines clinical and genetic variables and demonstrates good discrimination with an AUC of 85%. Removing the genetic variables results in a decrease in discriminative ability, as evidenced by an AUC of 0.79. This study demonstrates that it is possible to predict the response to MTX therapy in patients with recent-onset RA. Since patients in the model are treated with MTX monotherapy only, this may not be consistent with current principles of combination therapy, and subsequent studies have shown that it has an inadequate performance for the prediction of nonresponse to MTX in RA patients treated with combination therapies [35].

A similar predictive analysis was conducted in a study that established a discriminative model with good performance by combining genetic, metabolic, clinical, and lifestyle variables [19] (Table 1). The AUC of the model was 0.8 in both the derivation and validation cohorts. Another study not only predicted the efficacy of MTX in patients with RA, but also predicted its hepatotoxicity [20] (Table 1). The model showed moderate diagnostic accuracy for MTX efficacy (AUC = 0.84) and high diagnostic accuracy for liver toxicity (AUC = 0.91). However, there is currently a lack of external validation.

While the above models have indicated that genetic variables contribute to the improvement of the models’ discrimination, their involvement may indeed make the routine use of the models challenging. Taking this into account, Gosselt et al. [21] enhanced the applicability of the original model [19] by removing genetic variables and validating the model in cohort data from different regions (Table 1). The simplified model has an AUC of 0.75 and is successfully integrated in an online tool “Evidencio,” which can be available by https://www.evidencio.com/models/show/2191 [21]. The updated model is user-friendly and can be further validated and utilized in clinical practice to identify individuals who are insufficient responders to MTX. The goal is to promptly initiate additional biologic or JAK pathway inhibitor therapies for these individuals in order to minimize disease activity and slow disease progression.

A model involving imaging variables has been established [22] (Table 1). The study investigated if magnetic resonance imaging (MRI) or ultrasound (US) examination is useful in anticipating poor response to MTX, or future structural damage progression. The results indicate that the detection of inflammation by MRI or US is unrelated to predicting MTX response, but is rather associated with elements related to future disease progression.

### Models predicting insufficient response to tumor necrosis factor inhibitors (TNFi)

Upon conventional synthesis DMARDs (csDMARDs) such as MTX failure or loss of efficacy, the patients are switched to biologic DMARDs (bDMARDs), such as necrosis factor inhibitors (TNFi), for further treatment [6], but 30% of patients do not respond well to their initial TNFi therapy [36]. Therefore, the development of tools that can assist in providing practical guidance for the selection of candidate drugs for anti-tumor necrosis factor therapy is crucial.

A model was established to predict treatment response of RA patients to golimumab, a monoclonal anti-TNFα antibody [23] (Table 1). The AUC of this model is 0.648–0.809, when predicting 1-, 3-, and 6-month low disease activity or remission. A series of prediction matrix tools were created to facilitate the use of the model, which can be available at Rheumatology Online [23]. Although the model lacked external validation when it was published, follow-up research examined these tools in real-world RA patients undergoing anti-TNFα therapy and corroborated their effectiveness [37]. The data sources for establishing the model are large-sample studies across multiple countries, so they have great representativeness. Moreover, the readily accessible predictive factors facilitate the practical application of the model.

However, the study did not elucidate the biological mechanisms underlying this differential response to golimumab. Tao et al. [24] investigated the mechanisms of how RA patients respond differently to adalimumab or etanercept by analyzing gene expression and DNA methylation data, and established machine learning models to predict which therapy is effective for which patients before commencing therapy (Table 1). Adalimumab represents the initial fully human therapeutic monoclonal anti-TNFα antibody, whereas etanercept is a recombinant human TNF receptor (p75)–Fc fusion protein that functions as a competitive inhibitor of TNF [38]. This study suggests that response towards these two classes of TNFi is defined by the genetic and epigenetic differences between individual patients. However, whether the differential response to different drugs of monoclonal TNFi antibody or the inter-individual variability in response to a single drug is also determined by distinct genetic signatures remains a question that should be addressed in future studies.

### Models predicting insufficient response to rituximab or tocilizumab

Rituximab, anti-CD20 antibody, has been approved for use in RA patients who have failed or appeared intolerant to TNFi therapy [39]; however, approximately 30–40% of RA patients display a poor response to rituximab therapy [40]. A model composed of disease activity score (DAS) in 28 joints, interferon score, and DMARDs use was developed to predict non-response to rituximab in RA and exhibited an AUC of 0.82 [25] (Table 1). The use of prednisolone had a significant impact on the predictive performance of the model, which could be due to the impact of prednisolone on the interferon score. The mechanism underlying the association between a high interferon score and poor response to rituximab is yet to be elucidated. Future studies could optimize the model by elucidating this impact and its mechanism.

Another study established models for predicting treatment response to rituximab (AUC = 0.74), as well as response to tocilizumab, an anti-IL6R monoclonal antibody (AUC = 0.68), and multidrug resistance (AUC = 0.69), through in-depth histological and molecular analyses of synovial biopsies in RA patients [26] (Table 1). The post-treatment modifications in synovial gene expression and cell infiltration have revealed significant differences in the response/non-response mechanisms between rituximab and tocilizumab. The discovery of genes and cell types related to multidrug resistance is a significant development that could facilitate the creation of novel drugs for refractory patients who are unresponsive to available medications targeting conventional immune pathways. Further research can be conducted to elucidate the biological mechanisms underlying the differential response of patients to rituximab, tocilizumab, or multidrug resistance and to improve the performance of the model by optimizing the genetic variables.

## RA-CVD

### The possible pathogenesis and risk factors of RA-CVD

**Fig. 2:**
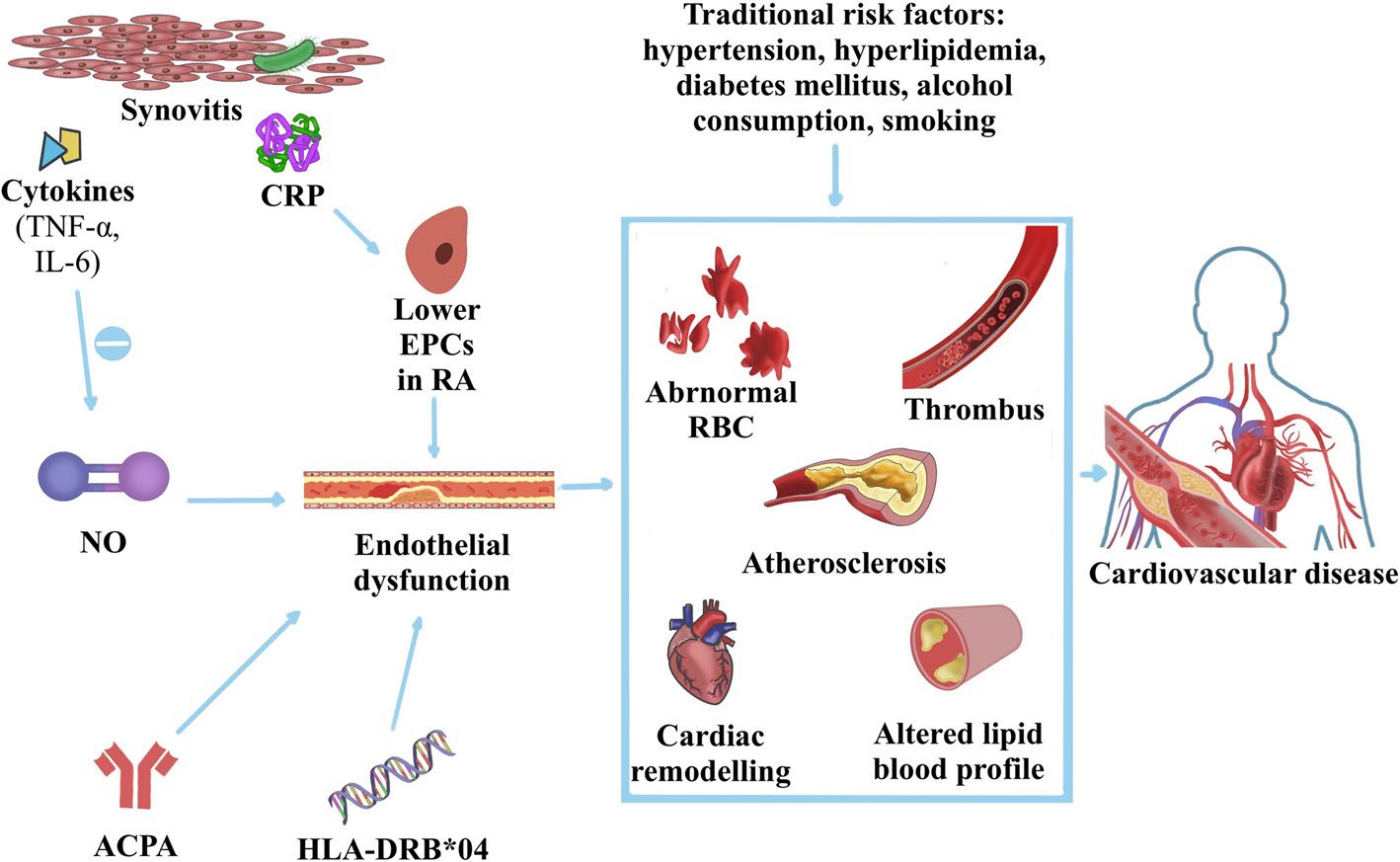
Schematic illustration of the pathogenesis of cardiovascular disease in rheumatoid arthritis. Levels of peripheral endothelial progenitor cells (EPCs) in RA are inhibited compared with general population, which could trigger the endothelial dysfunction (ED). C-reactive protein (CRP) can inhibit EPCs’ differentiation, survival, and function, which further leads to ED. The endothelial-activating cytokines presumably synovitis-derived, including interleukins (IL)-6 and necrosis factor inhibitors (TNF)-α, play important roles in endothelial damage since they inhibit the production of nitric oxide (NO), which, in turn, are responsible for maintaining a healthy endothelium. In addition, RA susceptibility genes human leucocyte antigen (HLA)-DRB1*04 and anti-citrullinated protein antibodies (ACPA) positivity also can contribute to ED. The endothelium plays a central role in atherosclerosis because it produces vasoactive substances including NO that act on the vascular tone and affects homeostasis between the circulating blood cells and the vessel wall. All of these factors, coupled with traditional risk factors for CVD such as hypertension, hyperlipidemia, diabetes mellitus, and smoking, may underlie pro-atherogenic and pro-thrombotic changes, the promotion of cardiac remodeling, alterations in lipid blood profiles, and changes to the morphology of red blood cells, which favor accelerated development of CVD in patients with RA

CVD is one of the most common complications of RA and the leading cause of mortality for patients [3], accounting for 30–40% of deaths [41], affecting approximately 2.4 to 18.6% of patients with RA [42]. Patients with RA have approximately 50% greater risk for CVD compared to the general population [43]. The main clinical manifestations of CVD are ischemic cardiomyopathy and congestive heart failure (CHF). CHF and myocardial infarction (MI) may occur twice as often in RA patients compared to the general population [44]. Due to the increased risk of MI, heart sudden death and stroke in patients with RA have been estimated to be twofold and 1.7-fold, respectively [45]. The pathogenesis of RA-ILD has not been fully elucidated, which may be associated with endothelial dysfunction (ED) and atherosclerosis due to inflammation-associated loss of elasticity of the vascular wall [46] (Fig. 2). Compared with the matched healthy control group, the levels of peripheral endothelial progenitor cells (EPCs) are lower in RA patients [47]. However, the lower the EPCs’ number, the worst the endothelial function [48], which could partly explain the ED observed in patients with RA. C-reactive protein (CRP) can inhibit EPCs differentiation, survival, and function, which eventually leads to ED [49]. The endothelium plays a central role in atherosclerosis because it produces vasoactive substances including nitric oxide (NO) that acts on the vascular tone and affects homeostasis between the circulating blood cells and the vessel wall [3]. Inflammation is the common link between atherosclerosis and RA, which can alter the balance between the production of NO and other vasoactive substances, causing ED and consequently promoting atherosclerosis [50]. The endothelial-activating cytokines presumably synovitis-derived, including interleukins (IL)-6 and TNF-α, play important roles in endothelial damage since they inhibit the production of NO, which, in turn, are responsible for maintaining a healthy endothelium [46]. In addition, an association has been found between ED and HLA-DRB1*04 shared epitope [51], the strongest genetic risk factor for RA. ACPA positivity also can contribute to the development of CVD and may induce subclinical atherosclerotic damage [52]. All of these factors, coupled with traditional risk factors for CVD such as hypertension, hyperlipidemia, diabetes mellitus, and smoking [53], may underlie pro-atherogenic and pro-thrombotic changes, the promotion of cardiac remodeling, alterations in lipid blood profiles, and changes to the morphology of red blood cells, which favor accelerated development of CVD in patients with RA [5, 46].

### Traditional CVD risk prediction models are not suitable for RA patients

Compared with traditional risk factors for CVD, patients with RA are more likely to cause CVD due to disease activity, ESR, CRP, RF, and ACPA [41, 54] (Table 2). Therefore, current methods for assessing CVD risk tend to underestimate the risk when applied to patients with RA. When the Framingham risk score and systematic coronary risk evaluation (SCORE) were applied to patients with RA, up to twofold risk underestimation was observed [55]. The risk of excess CVD is still attributed to inflammation, and current methods of assessing CVD risk do not account for RA patients who are chronically exposed to inflammatory environments [54]. To consider the effect of systemic inflammation of RA on CV risk, EULAR has suggested that the SCORE scoring system risk value be multiplied by 1.5 in RA patients who show at least two of the following: (1) RA disease of more than 10 years, (2) positive RF, (3) positive ACPA, and (4) presence of extraarticular manifestations [56]. It is, however, possible that even with the modified SCORE, a large number of RA patients still may not be identified and are at high risk for CVD [57]. The QRISK-2 scoring system includes RA as a risk factor for CVD, so there exist also expert consensus to recommend the use of QRISK-2 as a calculator for estimating the 10-year CVD risk of RA patients [58]. The study has shown cardiovascular risk age model and vascular age mode developed based on the SCORE model also has good performance when used in RA patients [59]. A limitation of these methods is that it treats all RA patients the same, regardless of the level of disease activity; therefore, there exists an urgent need for risk prediction models for CVD in RA patients.

**Table 2:** Risk factors for CVD and ILD in the general population and specific to rheumatoid arthritis

Disease	General population	RA
CVD	Age, sex, hypertension, hyperlipidemia, diabetes mellitus, alcohol consumption, smoking, obesity	ESR, CRP, RF, ACPA, RA duration, RA disease activity
ILD	Age, smoking, male, certain occupational, environmental exposures	RF, ACPA, RA duration, RA disease activity, radiographic joint damage

*Abbreviations*: *ACPA* anti-citrullinated protein antibodies, *CRP* C-reactive protein, *CVD* cardiovascular disease, *ESR* erythrocyte sedimentation rate, *ILD* interstitial lung disease, *RA* rheumatoid arthritis, *RF* rheumatoid factor

### CPMs of CVD for RA patients

An expanded risk score model for CVD in RA (ERS-RA) derived to predict 10-year probability of a CV event, such as MI, stroke, or CV-related death [27] (Table 1). To facilitate the use of the ERS-RA, a risk score calculator has been developed which can be downloaded at https://www.verityresearch.org/cvd-risk-calculator/ [27]. Although the model development data were derived from the cohort study in the USA, follow-up studies demonstrated the effectiveness of the ERS-RA in the European RA population [60]. The large sample size of the model’s data source and its validation in populations from different regions make the model highly reliable. Future research should focus on validating and continuously updating the model in populations of different races and regions.

A study [28] conducted a similar predictive analysis, establishing a prognostic model for CVD in RA patients by integrating clinical variables, laboratory indicators, and the multi-biomarker disease activity (MBDA) score (Table 1). The MBDA score evaluates the disease activity of rheumatoid arthritis by measuring 12 serum protein biomarkers and is associated with the risk of CVD in RA patients [61]. This may partially explain the additional CVD risk in RA patients caused by inflammation. Another study [29] reported a model to predict the occurrence of coronary heart disease (CHD) in RA patients (Table 1). This model differs from the above prognostic models in that it has the potential to screen out RA patients with concomitant CHD. It demonstrates superior performance in predicting RA-CHD compared to the Framingham risk score. The AUC for the model was 0.77, along with a 63.9% sensitivity and 77.2% specificity. However, its retrospective design and use of data from a single center highlight the need for continuous validation before its clinical use.

## RA-ILD

### The possible pathogenesis and risk factors of RA-ILD

**Fig. 3:**
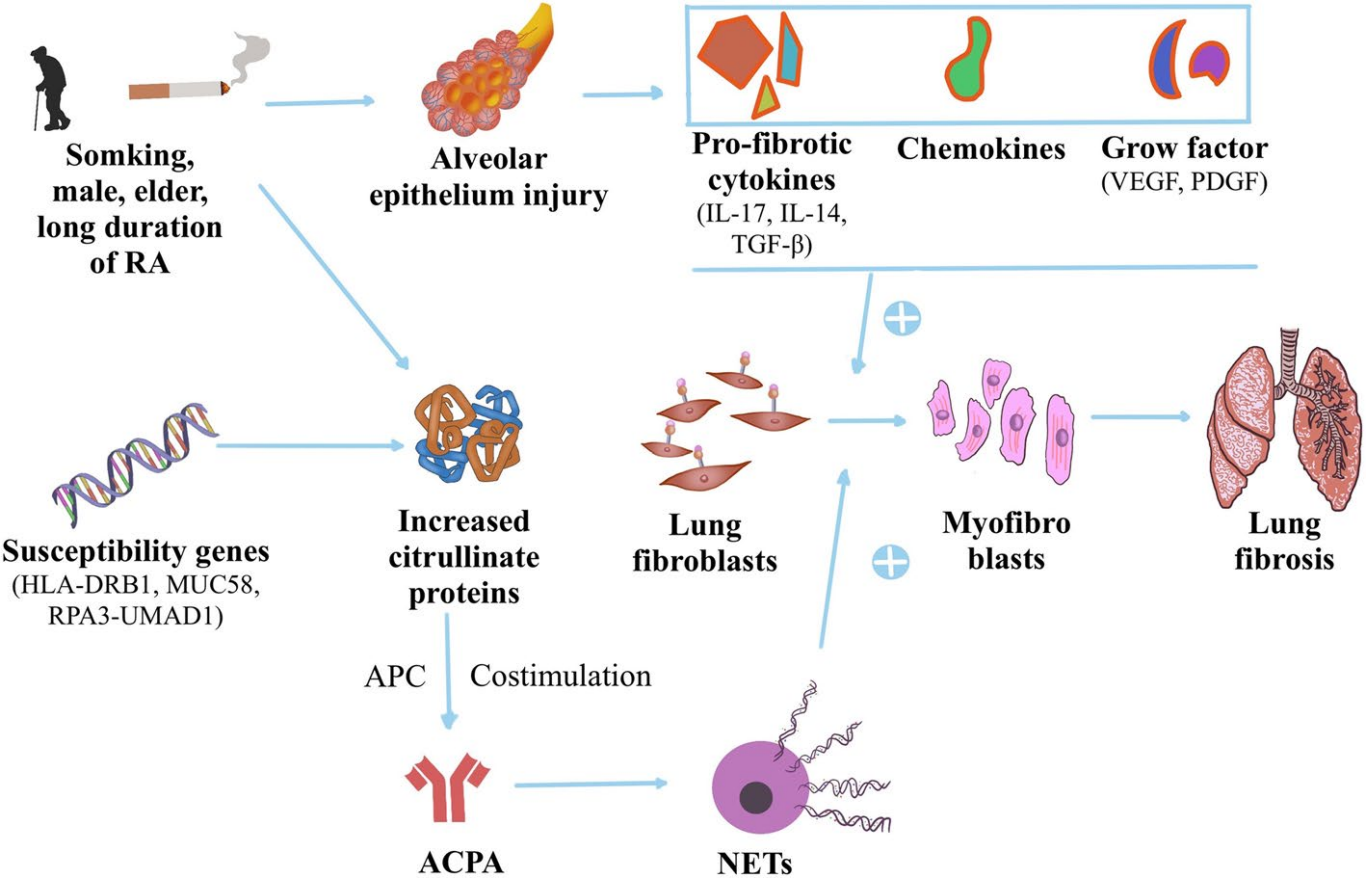
Schematic illustration of the pathogenesis of interstitial lung disease in rheumatoid arthritis. Alveolar epithelium injury from cigarette smoking characterized by cellular infiltration and release of pro-fibrotic cytokines including interleukins (IL)-17, IL-13, and transforming growth factor (TGF)-β, chemokines, and growth factors, such as vascular endothelial growth factor (VEGF) and platelet-derived growth factor (PDGF) that promote lung fibroblast proliferation and differentiation to myofibroblasts. Smoking also leads to the generation of citrullinated proteins in lung alveolar cell, which means higher levels of rheumatoid factor (RF) or anti-citrullinated protein antibodies (ACPA) can be found in the affected lungs of RA patients in the genetically susceptible individuals. Mechanistic study demonstrated ACPA is pathogenic and induces the release of neutrophil extracellular traps (NETs) which trigger the activation of lung fibroblasts to differentiate into myofibroblast, eventually leading to lung fibrosis formation. In addition, other risk factors including males, elder, and longer duration of RA, can also contribute to the development of RA-ILD. All this eventually leads to the development of lung fibrosis in patients with RA

The second major cause of death in patients with RA is respiratory disease, which occurs in 30–40% of patients [62]. ILD is the most common and severe manifestation of RA lung diseases [3], affecting approximately 2.2 to 10% of patients with RA [63, 64], and median survival after diagnosis keeps approximately 7 years [65]. Compared with general people, patients with RA have a much higher probability of developing ILD [66], but the possible pathogenesis of RA-ILD has not been fully elucidated, which can be summarized as the consequence of a combination of genetic, environmental, and autoimmune factors [67] (Fig. 3). The interaction of these factors contributes to the aberrant tissue response in the alveolar wall and pulmonary parenchyma, which include airways and alveolar epithelial cells, lung fibroblasts, and components of extracellular matrix [67]. MUC5B promoter variant rs35705950 [68] and rs12702634 at RPA3-UMAD1 [69] lead to genetic susceptibility in the West and East Asian populations, respectively. Smoking keeps the most significant risk factor for the development of ILD in patients with RA. Alveolar epithelium injury from cigarette smoking characterized by cellular infiltration and release of pro-fibrotic cytokines including IL-17, IL-13, and transforming growth factor (TGF)-β, chemokines, and growth factors, such as vascular endothelial growth factor (VEGF) and platelet-derived growth factor (PDGF) that promote lung fibroblast proliferation and differentiation to myofibroblasts [70]. Smoking also leads to the generation of citrullinated proteins in lung alveolar cells [71], which means higher levels of RF or ACPA can be found in the affected lungs of RA patients in genetically susceptible individuals. Mechanistic study demonstrated ACPA is pathogenic and induces the release of neutrophil extracellular traps (NETs) which trigger activation of lung fibroblasts to differentiate into myofibroblast, eventually leading to lung fibrosis formation [72, 73]. In addition, other risk factors including males, elder, and longer duration of RA can also contribute to the development of RA-ILD [64, 70] (Table 2).

### CPMs related to RA-ILD

The diagnosis of RA-ILD proves difficult, because approximately 5–10% of patients have significant clinical signs, and an additional 20–30% may have subclinical RA-ILD [66]. High-resolution computed tomography (HRCT) represents the gold standard for diagnosing the disease [74], but ILD can appear in any stage of RA, entailing the need for a systematic assessment of lung involvement. It is not advisable to use routinely HRCT for screening programs because of both high cost and X-ray exposure [75], and therefore, there exists an urgent need for a way to screen patients with RA who may develop ILD to target HRCT to patients who need it more. Lung auscultation represents an economical and radiation-free screening method for RA-ILD; the detection of the velcro crackle (VC) in lung sounds can effectively raise the suspicion of an ILD and speed up diagnosis [30]. However, this task largely relies on the experience of physicians and requires standardization in clinical practice.

Pancaldi et al. [30] investigated the problem of the automatic detection of VC in lung sounds and developed an algorithm called velcro sound detector (VECTOR) to detect the presence of VC in lung sounds recorded by electronic stethoscope to infer the presence of ILD in RA patients (Table 1). The VECTOR demonstrates higher accuracy than clinical physicians in diagnosing RA-ILD. When VECTOR was validated in different populations, it showed a diagnostic accuracy of 83.9% and a sensitivity and specificity of 93.2 and 76.9%, respectively [76]. In general, the identification of VC has always been qualitative and subjective, but the proposal of VECTOR has the potential to transform it into a quantitative and objective process. Because the auscultation of lung sounds is inexpensive and non-invasive, VECTOR can be used as a routine screening tool for RA-ILD.

A study [31] analyzed the influencing factors of RA-ILD and constructed a diagnostic model with good discriminative ability (Table 1). The study included traditional Chinese medicine (TCM) variables as predictors, meaning that variables from complementary and alternative medicine may also contribute to model development. Another study [32] reported a prognostic model for RA-ILD (Table 1). Unlike the RA-ILD screening model mentioned above, this prognostic model provides a predicted probability of death after 90 days of acute exacerbation (AE)-RA-ILD. This study identified forced vital capacity (FVC) within the 12 months preceding AE and the ratio of arterial partial pressure of oxygen to fraction of inspired oxygen (PaO2/FiO2) during AE onset as independent predictive factors for mortality, which may contribute to the prognostic management of RA-ILD.

## Discussion

This review aims to summarize CPMs related to RA, RA-CVD, and RA-ILD, in order to provide reference and evidence for the early diagnosis and treatment of these diseases and personalized medicine for patients. Moreover, the pathogenesis and risk factors of RA-CVD and RA-ILD are summarized. Recently, some literature has provided separate overviews of the risk factors and pathogenesis of RA-CVD [77] and RA-ILD [78], which bear similarities to our research. However, in addition to this, our study highlights the development of predictive models for these diseases. Interestingly, these studies have mentioned the necessity of screening RA patients for CVD or ILD, but currently, there is a lack of effective screening methods or tools in routine clinical practice, which is the problem our study aims to address.

The pathogenesis of RA-CVD and RA-ILD have not been fully elucidated, and genetic characteristics and inflammation may play an essential role in these processes [3]. Disease activity and systemic inflammation are the most common implicated non-traditional cardiovascular risk factors in inflammatory joint diseases [79]. Research by Solomon et al. [80] has proved that there was a 21% reduction in CVD risk for every 10-point reduction of the Clinical Disease Activity Index (CDAI) in patients with RA. Similar to RA-CVD, RA-ILD risk increased by 35% for each additional unit of DAS28 [81]. ACPA, as the most representative autoimmune antibody for RA, also seems to be involved in the development of various comorbidities. ACPA can lead to the development of CVD by contributing to ED in RA patients [29, 49] (Fig. 2). In the process of RA-ILD, NETs were released by the impact of ACPA, which trigger the activation of lung fibroblasts to differentiate into myofibroblast, eventually leading to lung fibrosis formation [72, 73] (Fig. 3).

CPMs use information about a patient at baseline to predict the risk of a current (diagnostic) or future (prognostic, e.g., non-response/adverse events) clinical event [82], which can not only provide high-quality evidence for evidence-based medicine [83], but also serve as a favorable tool for the application and popularization of precision medicine. With the advent of the era of precision medicine, clinical prediction models are increasingly used in medical diagnosis and treatment decisions, patient prognosis management, and public health resource allocation, so their value is becoming more and more important [9].

At present, the CPMs that predict drug response in the treatment of RA mainly concentrate on MTX and bDMARDs. It is worth noting that genetic variations have a certain impact on the therapeutic response to MTX [18–20], adalimumab [24], etanercept [24], rituximab [26], or tocilizumab [26]. The high cost of genetic testing may present a challenge for the routine use of the models. In future studies, it would be of interest to perform comprehensive cost–benefit analyses, examining the cost of genetic testing in relation to long-term medical treatment expenses and clinical and functional outcomes. There is currently no model that is effective in predicting the treatment response of JAK inhibitors (such as tofacitinib, baricitinib, and upadacitinib). JAK inhibitors are new targeted synthetic DMARDs used in the treatment of RA and are an important approach for treating the condition. However, their safety has been the subject of controversy [84]. Therefore, future research should not only focus on predicting the therapeutic response of these drugs but also consider their potential side effects and make predictions accordingly.

CVD is the most urgent and serious complication of RA because it is strongly associated with an increased risk of death [44]. In addition to traditional and RA-specific risk factors for CVD, biomarkers of cardiac dysfunction, including N-terminal pro-brain natriuretic peptide (NT-proBNP) and cardiac troponin T, have also been reported to predict CVD risk and mortality in RA patients [46]. It is noteworthy that although ED plays a crucial role in the development of RA-CVD [46], it has not been included in the current models as a predictive factor. This may be attributed to the fact that current research mainly focuses on traditional CVD risk factors and additional risk factors caused by systemic inflammation in RA, without delving into the underlying mechanisms of RA-CVD. This may explain why these models only have moderate discriminative ability, with an AUC of less than 0.8 [27–29]. Currently, there are several feasible approaches to assess ED, such as non-invasive examinations (flow-mediated dilation, subcutaneous adipose tissue thickness, and carotid intima-media thickness) as well as biomarkers (ischemia-modified albumin, pentraxin-3, E-selectin, endothelin-1, von Willebrand factor, endothelial microparticles, and EPCs) [85]. Among them, EPCs, E-selectin, and von Willebrand factor have been measured in RA patients and are associated with RA-CVD [86]. Identifying the optimal method for measuring endothelial function, which can be used to predict the risk of RA-CVD, is a crucial area for future research.

In 20–30% of patients with RA, a pulmonary complication is the first manifestation, rather than joint symptoms [87]. Therefore, some scholars also proposed another possible pathogenesis of RA-ILD that idiopathic pulmonary fibrosis-like pathology triggers an immune response to citrullinated proteins that promotes articular disease indicative of RA [88]. Interestingly, although RA is more common in females, with a female-to-male sex ratio ranging as high as 4:1 [89], RA-ILD is more prevalent in males, with a male-to-female ratio of 2:1 [67]. Therefore, men with RA should be highly alert for the development of ILD, and smoking cessation should be put on the agenda as early as possible. Smoking as a common risk factor for RA and RA-ILD, recent studies have shown that smoking may exhibit a threshold effect in its relationship with RA-ILD that smoking 30 pack-years or more was associated with a sixfold increase in RA-ILD risk, whereas smoking under this threshold was not associated with increased risk [90]. Therefore, it is not enough to focus on whether patients smoke, and future studies should further explore the relationship between the number of cigarettes smoked and RA-ILD. Several new biomarkers can enhance the detection of RA-ILD, including matrix metalloproteinase, surfactant protein D, and pulmonary and activation-regulated chemokine [91], which may be promising for the development of new predictors in future research.

It is noteworthy that only a few models have been updated in subsequent clinical practices in this review [11, 19]. Therefore, in addition to the development of new predictors and models, validation and updates of existing models should also be an area of future research focus. Every study has limitations; this study is no exception. Firstly, the comorbidities only focus on CVD and ILD and were not all-inclusive; some important comorbidities such as osteoporosis depression and malignancies were not included. Secondly, we only evaluated the predictive ability of the models and did not assess whether their methods are reliable.

## Conclusions

In summary, the pathogenesis of RA-CVD and RA-ILD prove undoubtedly complex. Inflammation, disease activity, and specific autoimmune antibody are all inextricably associated with the development of these complications. We attempt to summarize the possible pathogenesis of these diseases that the combination of inflammation, autoimmune response, disease activity, and related traditional risk factors under the impact of susceptibility genes can lead to ED, and maturation of myofibroblasts, and ultimately to the occurrence in RA patients of CVD and ILD, respectively.

CPMs have the advantage of early detection of complications and prediction of drug response even in RA with complex pathological mechanisms. Therefore, in addition to new drug development, it is equally important to predict the effective response of RA patients to therapeutic drugs and early identification of patients who are prone to various complications. We hope that the future development of CPMs will take us from the current trial-and-error drug prescribing and into an emerging era where the selection of the optimal drug is based on pre-treatment predictions.

## Data Availability

Not applicable.

## Abbreviations

ACPA: Anti-citrullinated protein antibodies
ACR: American College of Rheumatology
APC: Antigen-presenting cells
AUC: Area under the curve
cfPWV: Carotid-femoral pulse wave velocity
CHD: Coronary heart disease
CPMs: Clinical prediction models
CRP: C-reactive protein
CVD: Cardiovascular disease
DAS: Disease activity score
DMARDs: Disease-modifying antirheumatic drugs
ECG: Electrocardiographic
ED: Endothelial dysfunction
EPCs: Endothelial progenitor cells
ESR: Erythrocyte sedimentation rate
FVC: Forced vital capacity
GC: Glucocorticoid
HAQ: Health assessment questionnaire
HDL: High-density lipoprotein cholesterol
HRCT: High-resolution computed tomography
IL: Interleukins
ILD: Interstitial lung disease
MBDA: Multi-biomarker disease activity
MI: Myocardial infarction
MMP: Matrix metalloproteinase
MTX: Methotrexate
NETs: Neutrophil extracellular traps
NR: Not reported
OP: Osteoporosis
PGA: Patient global assessment
RA: Rheumatoid arthritis
LDL: Low-density lipoprotein cholesterol
RAI: Ritchie articular index
RF: Rheumatoid factor
SCORE: Systematic coronary risk evaluation
SJC: Swollen joint count
TG: Triglyceride
TGF: Transforming growth factor
TJC: Tender joint count
TNFi: Tumor necrosis factor inhibitors
US7: 7-Joint ultrasonic erosions score
VC: Velcro crackle
VECTOR: Velcro sound detector
VEGF: Vascular endothelial growth factor
